# Organoids and the genetically encoded self‐assembly of embryonic stem cells

**DOI:** 10.1002/bies.201500111

**Published:** 2015-12-15

**Authors:** David A. Turner, Peter Baillie‐Johnson, Alfonso Martinez Arias

**Affiliations:** ^1^ Department of GeneticsUniversity of CambridgeCambridgeUnited Kingdom

**Keywords:** development, organoids, positional information, reaction‐diffusion, self‐assembly, self‐organization

## Abstract

Understanding the mechanisms of early embryonic patterning and the timely allocation of specific cells to embryonic regions and fates as well as their development into tissues and organs, is a fundamental problem in Developmental Biology. The classical explanation for this process had been built around the notion of positional information. Accordingly the programmed appearance of sources of Morphogens at localized positions within a field of cells directs their differentiation. Recently, the development of organs and tissues from unpatterned and initially identical stem cells (adult and embryonic) has challenged the need for positional information and even the integrity of the embryo, for pattern formation. Here we review the emerging area of organoid biology from the perspective of Developmental Biology. We argue that the events underlying the development of these systems are not purely linked to “self‐organization,” as often suggested, but rather to a process of genetically encoded self‐assembly where genetic programs encode and control the emergence of biological structures.

**Abbreviations**
EB: embryoid body
(E)SC: (embryonic) stem cell
FP: floor plate
iPSC: induced pluripotent stem cell

## Introduction

**Figure 1:**
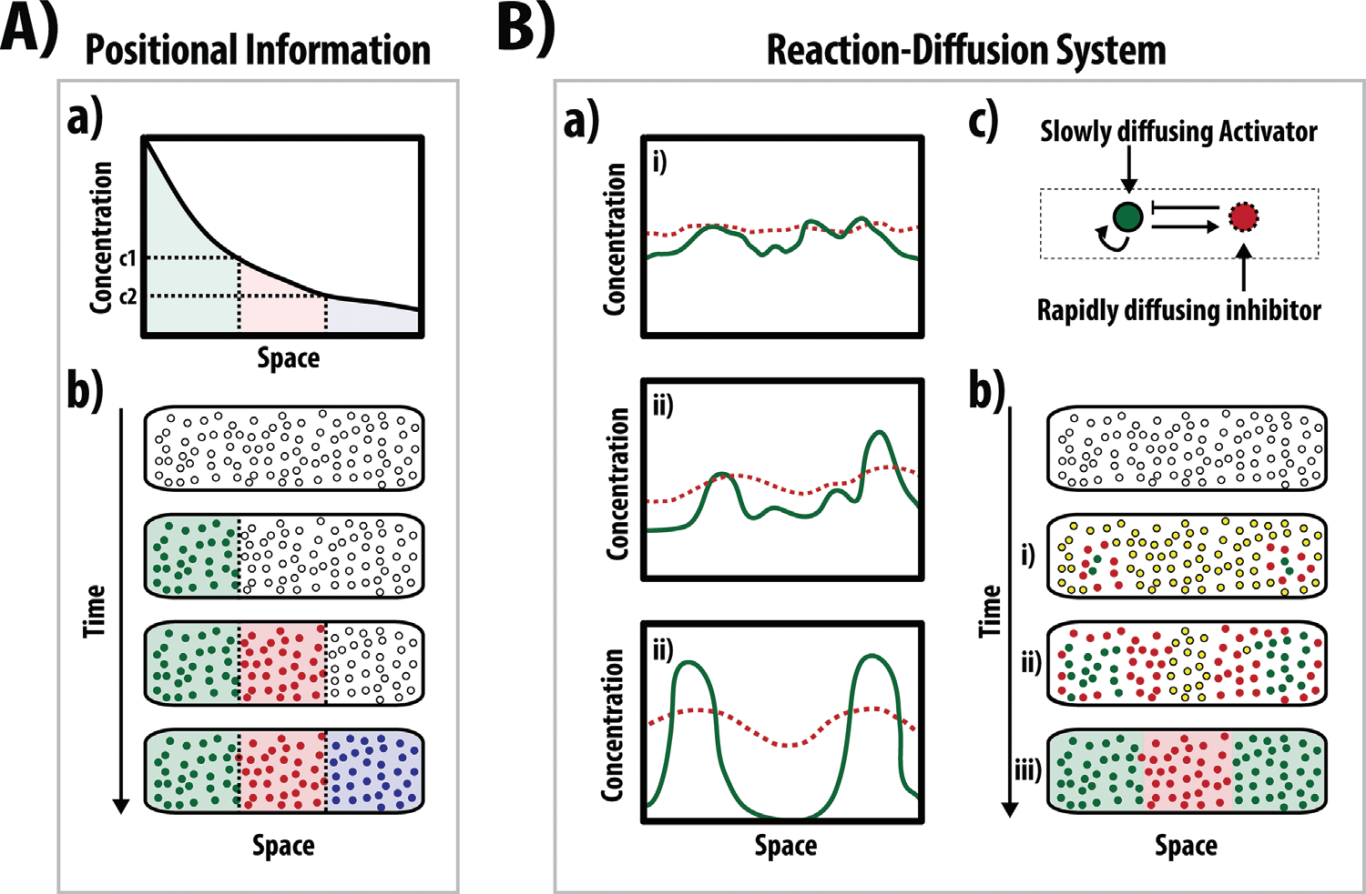
Patterning tissues through Positional Information or a Reaction‐Diffusion System. **A:** *Patterning through Positional Information*. Secretion of a Morphogen from a fixed source results in a graded distribution of the signal through the tissue. This signal is interpreted by cells where their fate depends on defined concentrations of the Morphogen (c1, c2). **B**: *Patterning through Reaction‐diffusion (R‐D) systems (Turing)*. In Turing's R‐D model, two genes interact where one activates its itself (green) as well as its inhibitor (red; **c**). Critically, since the activator diffuses slowly with respect to the inhibitor, the inhibitor is unable to provide enough negative feedback to counter the autoinduction of the activator at the point of initiation. This results in sharp peaks centred around regions of inhibition (**a‐ii**, **b‐ii**). As the levels of inhibitor decrease around these local maxima, other peaks can form until the expression of these genes dynamically alters to produce a regular oscillatory pattern (**a‐iii**, **b‐iii**). The wavelength of these oscillations depends on the size and shape of the tissue being patterned, where the concentration of specific substances produced by these oscillations may determine the specific fates a tissue will adopt. Figure part adapted from 91 and 97.

Embryonic development transforms a single celled zygote into a collection of multicellular tissues and organs arranged into structures we call organisms. A key element in this transformation is the ordered generation of cellular diversity which depends on the progressive allocation of cells to specific fates and their self‐assembly into three dimensional structures according to emergent rules encoded in those fates. This process depends on programs encoded in, and decoded by, signaling and transcriptional networks. For the last 50 years our understanding of how these molecular devices organize cells in space and time has been dominated by the notion of *Positional Information* (Fig. 1A). Introduced by Lewis Wolpert in 1969 1, Positional Information states that in a developing organism cells acquire fates by “reading and interpreting” molecular instructions encoded in diffusible substances which, following a terminology introduced by Alan Turing, are known as *Morphogens* 2. A most important element of Positional Information is the notion that Morphogens diffuse from a fixed source across a cellular field thus creating a concentration gradient with different concentrations evoking different responses in the underlying cells i.e. the position of a cell relative to the source of the Morphogen is transformed into a fate. Genetic analysis of pattern formation in *Drosophila* identified genes whose products could be cajoled into mediating Positional Information through a classic Wolpertian mechanism 3, a notion that was later extended to other organisms. However, the observation that Wingless, a leading member of the Wnt gene family and an influential Morphogen, does not work at a distance in *Drosophila* 4, 5 and that time of exposure to, and concentration of, a Morphogen are interchangeable variables for patterning fields of cells 6, has invited a reflection on the role of gradients in pattern formation. An alternative to Positional Information preceded Wolpert's ideas and was put forward by Alan Turing. In his seminal paper of 1952 he showed how, under certain conditions, random heterogeneities in chemically interacting diffusible substances could generate patterns without a pre‐existing organisation (Fig. 1B) i.e. they could act as agents of self‐organization 2. A few years later, in an independent study, Gierer and Meinhardt proposed a formally equivalent solution to the problem of spatial patterning in biological systems 7. Turing's ideas, a theoretical proof of principle, were difficult for biologists. This together with the geometric and intuitive design of *Drosophila* development as well as the appeal of the Wolpertian Morphogen metaphor to explain the patterning of the vertebrate limb 8, 9, led developmental biologists to embrace *Positional Information* rather than *Turing‐based* mechanisms as a basis for the patterning of cells during developments.

The last few years have produced a large number of observations that cells can organise themselves into recognisable patterns without a fixed reference. These observations are difficult to relate to the classical views of pattern formation and suggest that, perhaps, the classical notion of *Positional Information* is in need of a revision. The ability of cell ensembles to organize themselves into patterns resembling those that arise in embryos finds a surprising extreme in the experimental ability to coax stem cells into building different structures, from an eye cup 10 to an intestine 11. In particular, embryonic stem cells (ESCs) can be steered into specific tissues and organs with surprising ease. This ability has been referred to as *self‐organization* and, by implication, evokes notions of Turing‐like mechanisms (Box 1). Whereas these observations have been hastily discussed in the context of regenerative medicine, it may be the case that they are telling us more about both Development itself and how we shall be able to use this information practically. In this essay, we shall discuss these novel observations from the perspective of Developmental Biology. We shall question the notion that organoids exclusively represent examples of self‐organization and suggest that they reveal interactions between cells and underlying genetic programs that encode emergent properties of developmental systems.

Box 1 **Definition of Terms**

*Genetic Program*: In Developmental Biology, a genetic program is a temporal sequence of changes of state of a cell or cell population, brought about by the decoding of a temporal order of gene expression scripted in the genome.

*Self‐Assembly*: The formation of an ordered structure from non‐equivalent parts as a system moves towards equilibrium.

*Self‐Organization*: The spontaneous emergence of order or asymmetry from an initially homogeneous starting population that occurs in an energy‐dependent manner.

*Genetically‐Encoded Self‐Assembly*: A genetic program that contains cell autonomous instructions as well as signalling events which can induce emergent properties.

## Anterior neural as a primary fate in stem cells and embryoid bodies

Stem cells (SCs) have the defining characteristics of self‐renewal and the ability to differentiate into specialized cell types. Generally there are two classes of SCs, embryonic (ESC) and somatic (adult), the former being derived from the inner cell mass (ICM) of the pre‐implantation embryo 12, 13 are able to generate all the tissues of the embryo proper, whilst the latter sustain the homeostasis and fuel repair mechanisms of differentiated tissues and organs 14. The ability of SCs to be maintained in culture and their propensity to differentiate into the different cell types of the developing organism has resulted in their use as a model system for investigating biological processes such as early developmental events, self‐organization, tissue homeostasis, and repair.

**Figure 2:**
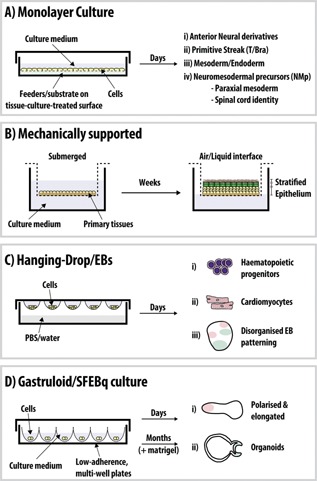
Comparison of culture methods. Schematics of the typical culture methods utilised for the differentiation of SCs. **A**: Cells grown as a monolayer on a bed of feeders or surfaces coated with substances such as gelatin or fibronectin. In the case of ESCs, specific culture conditions can direct their differentiation towards anterior neural (**i**), a primitive streak (PS) population (e.g. T/Bra‐expressing cells 66; **ii**), derivatives of the germ layers (**iii**) and a neuromesodermal progenitor (NMp) population for axial tissues such as the spinal cord and paraxial mesoderm 74, 98. **B**: Mechanically supported culture allows the further differentiation of primary tissues such as human keratinocytes. Upon contact with an air‐liquid interface and over a period of weeks, cells differentiate and self‐assemble to form a fully stratified tissue (adapted from 15). **C**: Embryoid bodies (EBs) can either be generated on low‐adherence tissue‐culture plastic or through hanging drop culture (pictured). In the latter case, droplets of ESCs are suspended above PBS or water and cultured for a number of days. Haematopoietic progenitors (**i**) 23 and cardiomyocytes 22 (**ii**) have been produced through EB culture. EBs typically show disorganised gene expression (**iii**), however polarised, elongated structures have been formed by this method using low numbers of EC cells 75. **D**: More modern techniques producing “*Gastruloids*” (**i**) and the serum‐free floating culture of embryoid‐body‐like aggregates with quick reaggregation (SFEBq) 27 (**ii**) have been successful in generation of structures that mimic a number of early developmental processes (axial elongation, polarisation; (**i**) as well as the generation of self‐assembling and patterned organoids such as the optic cup (**ii**). In the case of the latter organoids, cells are usually embedded in Matrigel and occasionally transferred to bacterial dishes once aggregation has occurred. See Table 1 for details on the culture methods and time for organoid formation.

Classically, experiments using SCs have relied on two dimensional culture techniques, where cells are grown on plastic dishes as a monolayer (Fig. 2A). ESCs for example, are traditionally cultured on either a bed of feeder cells (which provide a number of factors that maintain their pluripotency e.g. LIF in the case of ESCs) or adherent substrates (such as fibronectin or gelatin) (Fig. 2A). Although this two dimensional culture method has been exceptionally useful as a foundation for understanding many cellular processes, it cannot recapitulate the three dimensional environment cells are exposed to in vivo 15. Allowing cells to grow in three dimensions reveals a potential for them to assemble spatially organized patterns (Fig. 2B–D). Early pioneering studies from the laboratory of Howard Green and colleagues showed how cultured primary human skin cells on a bed of irradiated 3T3 cells could form a stratified squamous epithelium 16, presumably derived and maintained by the SCs present within the primary tissue 17. Furthermore, mechanically supported cultures of primary keratinocytes from the skin or oesophagus (Fig. 2D) can generate fully stratified, organized epithelia upon their making contact with an air‐liquid interface 15, 18, 19 (Table 1). The mechanical support provided by artificial matrices and scaffolds also allows primary endothelial cells to generate blood vessels with tissue architecture not dissimilar from their in vitro situation 20.

**Table 1:** Comparison of the culture techniques, generation time, and plating densities of organoids

Organoid	Origin	No. Plated cells/tissues	Method/Comments	Time to generation (days)	Ref.
Anterior					
Cortical Neurones	m/hESCs	3 × 10^3^ cells	SFEBq; low‐adhesion U‐bottomed plates	10–20	27
Optic cup	mESCs	3 × 10^3^ cells	SFEBq, Matrigel embedding; low‐adhesion U‐bottomed plates	∼9	26
	hESCs	9 × 10^3^ cells	SFEBq low‐adhesion V‐bottomed plates; Matrigel embedding day 2; transfer to petridish day 12;	∼24	10
			SFEBq; low‐adhesion plates; Matrigel embedding	14–24	
Inner Ear	mESCs	3 × 10^3^ cells			93, 94
Cerebral	mESCs	2 × 10^3^ cells	EBs generated in low‐adhesion U‐bottomed plates; embedded in Matrigel and cultured in spinning bioreactor	30–75	39
	hESCs/iPSCs	4.5 × 10^3^ cells			
Neural Tube	mESCs	1 cell	Cells (5 × 10^4^ cells) in N2B27 embedded in Matrigel, spread evenly over glass‐bottomed MatTek dishes; organoids form from single cells	<10	83
The viscera					
Intestine	Crypts (m)	500 Crypts	Matrigel embedding; Single LGR5^+^ forms organoids; enhanced with Paneth cell co‐culture	8–14	11, 47
	LGR5^+^ SC (m)	1 cell			
	LGR5^+^ + Paneth (m)	500 cells each			
	hESCs/iPSCs	50 spheroids	Monolayer differentiation towards hindgut; formed spheroids embedded in Matrigel	14–28	51, 52
Colon	Crypts (m, h)	500 Crypts	Matrigel Embedding; single LGR5^+^ SCs can form organoids if anoikis is inhibited in first 2 days	7–10	48
	LGR5^+^ SC (m)	1 × 10^3^ cells			
Stomach	Gastric glands (m)	100 glands	Matrigel embedding	7–10	49
	LGR5^+^ SC (m)	50 cells			
	hESCs/iPSCs	50 spheroids	Monolayer differentiation towards posterior foregut; spheroids embedded in Matrigel	28	57
Lung	hESCs	50 spheroids	Monolayer differentiation towards anterior foregut; spheroids embedded in Matrigel	65–110	53
Kidney	hESCs	1 × 10^6^ cells	Monolayer differentiation towards intermediate mesoderm, dissociation and culture in Air‐Liquid interface after 18 days in culture	4	43
Liver	hiPSCs	1 × 10^6^ cells	Monolayer differentiation towards endoderm; co‐culture with HUVECs and hMSCs on Matrigel	4–6	46
	Biliary Ducts (m)	100 glands	Matrigel Embedding	7	95
	LGR5^+^ SCs (m)	1 cell	Matrigel Embedding	19	95
Embryo					
‘Gastruloids’	mESCs	∼400 cells	Cells plated in low‐adhesion, U‐bottomed plates	4–5	73, 74, 96
Other					
Skin	Primary keratinocytes (h)	3 × 10^5^ cells	Air‐liquid interface culture	∼ 21	19
Oesophagus	Oesophageal fibroblasts (m/h)	2.5 × 10^5^ cells	Fibroblasts embedded in collagen/matrigel; Oesophageal keratinocytes (4 × 10^5^) added after seven days; Air‐liquid interface culture	11–13	18
Blood vessels	HUVECs	4.5 × 10^4^ cells	Cells seeded into collagen microvessels (mechanical support)	7–14	20

Information on selected organoids from a number tissues, broadly grouped into three categories, is given for the tissue origin (e.g. ESC, adult SC, tissue fragments etc.), number of cells or individual cellular units (crypts or cell spheroids) used to generate the organoid and the time taken to form the organoid structure. Time to formation is taken as the amount of time forming organoid structure, not the total time in culture. This is particularly important in the case of the visceral organoids from SCs, where cells are first directed to specific lineages *before* Matrigel embedding and organoid formation. ESCs: embryonic stem cells; SCs: stem cells; (h): human; (m): mouse; SFEBq: serum‐free floating culture of embryoid‐body‐like aggregates with quick reaggregation; HUVECs: Human Umbilical Vein Endothelial Cells; hMSCs: human Mesenchymal Stem Cells.

Culturing ESCs in high density, non‐adherent, suspension culture, gives rise to aggregates that form three dimensional structures termed *Embryoid Bodies* (EBs; Fig. 2C) 21. ESCs differentiated in this manner, typically requiring many thousands of ESCs which grow in a largely disordered manner, are able to progress towards further stages of early embryo development 21. This therefore provides an attractive system for deriving a number of embryological cell types, some of which are not easy to obtain in adherent culture e.g. blood 22 and cardiac 23. Sometimes, sorting of cell types with different characteristics can be observed within a single EB as in the case of endoderm 24 (Fig. 2B). Whereas the emerging organoid field also relies on three dimensional suspension culture, these organoids are typically studied as intact structures throughout their development rather than continued culture in two dimensions following initial EB formation.

However, though sometimes pockets of spatial organization can be found in EBs 25, these are not structured in the manner of the organs in embryos. Building on this observation and making use of fundamental principles of developmental biology, Sasai and his team were able to generate optic cups from ESC aggregates in the absence of external mechanical inputs in around nine days 26. During the first five days of culture, approximately 80% of the aggregates form a retina anlagen at a specific position with an almost perfect organization. In doing so, it is conceivable that the ESCs generate an underlying pattern through a self‐organizing process. However, Sasai and colleagues stressed that the contribution of self‐organization to this structure is the information required for self‐assembly – as soon as the cells start to express *Rx* (a marker of retina tissue), they begin to assemble themselves, changing their properties in a genetically predictable manner. Over the following 4 days, this emerging tissue arranges itself into a well‐formed optic cup through mechanically and biochemically imposed changes to the tissue. These observations allowed the authors to identify self‐organization as a means to form highly ordered structures from an unpatterned cellular ensemble, neatly describing the origin of the anlagen 26. In the same cultures it is often possible to observe the emergence of anterior brain structures: through tweaking of the culture conditions, diverse structures of the forebrain, which are the source of the optic primordia are generated efficiently, although not predictably 26. Interestingly, the essence of these protocols tends to be the suppression of most external signals 27 and reflect a (developmental) tendency of the ESCs to develop these structures 28, 29, 30, 31. These observations are presaged in classical experiments with *Xenopus* embryos in which animal caps, if left in simple medium, differentiate into forebrain with the occasional emergence of eye tissues 32, 33, 34, 35 suggesting that the anterior neural fate might be a universal primary fate in development.

The emergence of a complex structure such as an eye cup from a collection of cells without an external reference is, at first sight very surprising. One possible explanation for this observation is that within the large numbers of cells undergoing anterior neural development in the culture, one or a few of them might, just by chance, activate the eye cup genetic program in an environment which amplifies it and takes it to term. This situation would be reminiscent of the emergence of compound eyes in *Drosophila* upon ectopic activation of the Eyeless/Pax6 transcription factor 36. In these experiments, the result of this misexpression is the production of well‐formed and histologically complete compound eye structures. However while the ectopic expression occurs in large spatial domains across whole imaginal discs, the ectopic eye tissue occupies a smaller domain, generally in the same place within each disc, and tends to form contained structures. This observation, which has also been made for the emergence of ectopic wings 37, indicates that the development of specific structures relies not only on the activation of a specific cell‐autonomous genetic program, but also on the convergence of specific signals and mechanical inputs that restrict the potential to develop the genetic program. The ectopic transcription factor can only act where there is a constellation of signals which will drive its activity 37, 38. It is possible that the same happens with the mouse ESC‐derived eye cups, though in this case it is a stochastic event within a very large cell population in culture and highlights the relationship between environment, transcription and, probably mechanics.

The emergence of eye cups from ESCs contrasts with the events associated with the in vitro derivation of regionalized cerebral cortex in organoid culture 39. In this case, the whole structure develops into a mixture of locally organized tissues that are proposed to interact with one another, but there is no global organization as in the previous examples. Where the results of the neural cyst cultures showed that the organoids are capable of generating a rudimentary axis, there is no overall co‐ordination in the growth of these cortical tissues, perhaps indicating that local tissue interactions may play a greater role in patterning in this case. It is worth noting that the period of culture is much longer in these aggregates than the organoids described so far (Table 1); this may be partially a property of the human stem cell system but it could also reflect a much longer period of self‐assembly, following local tissue self‐organization.

## Mesendodermal organoids can emerge from adult and embryonic stem cells

A number of in vitro models have been established which generate organoids of visceral tissues either directly from pluripotent SCs (ESC and iPSC) or from adult SCs, primary cells, dissociated tissues or organ slices 15 (Table 1). To date, the pancreas 40, kidney 41, 42, 43, 44, thyroid 45, liver 46, various regions of the gastrointestinal (GI) tract 11, 47, 48, 49, 50, 51, 52 and respiratory system 53, 54 have been described. Similar to the examples for anterior structures (above), the time taken to generate these structures can vary following the initial plating event. Once more the structures are said to develop primarily through self‐assembly but the self‐assembling of the tissues is subtly different compared with anterior, neural organoids (above) as well as between visceral organoids formed from either embryonic or adult stem cells.

### Visceral organoids from adult tissue

During the generation of GI‐tract organoids, the initial approaches relied on isolating crypts from either the intestine 11, colon 48, or stomach 49 and embedding them in matrigel (Table 1). As these crypts contain LGR5‐expressing crypt base columnar (CBC) cells, the adult SC population, which are able to re‐generate all epithelial lineages of its respective tissue 55, it is therefore no surprise that over time, the crypts containing these cells are key to the regeneration of the tissue structure in vitro. Indeed, as the three dimensional culture takes shape, further crypt domains are generated within the matrigel culture, forming an organoid with multiple crypts interspaced by a villus‐like epithelium that surrounds a central lumen by approximately fourteen days. Generally, the *initial* culture period of these organoids from the isolation of the adult SC population to the initiation of organoid development takes approximately eight days in culture, much more rapid than those observed with most anterior structures (Table 1). This rapid development may be attributed to the initial patterning and positional information that is inherent in the isolated crypts, where the signalling environment is permissive for their self‐assembly. In support of this, whereas single LGR5^+^ cells can only generate organoids approximately 6% of the time 11, co‐culture of LGR5^+^ cells with Paneth cells (500 cells each) is much more efficient 47, suggesting that the heterogeneities created through the co‐culture of these cells may facilitate the initial self‐organizing pattern formation: e.g. Turing‐like mechanisms (Fig. 1B) may allow these self‐assembling processes to progress more efficiently as the initial asymmetry is already established.

### Visceral organoids from ESCs

The generation of organoids for visceral tissues directly from embryonic or induced pluripotent SCs requires a different approach. In these conditions, pluripotent SCs are guided sequentially towards lineages that are the primordia for their tissues by supplying in vitro the chemical signals that would be received during development (i.e. directed towards the definitive endoderm for tissues derived from the primitive gut tube 56 or towards the primitive streak/mesoderm for derivatives of the kidney 42, 43). For example, differentiation of ESCs towards the definitive endodermal lineage with a 3 day treatment of high dose Activin, followed by a further 4 days of specific differentiation signals can guide them towards posterior foregut (stomach 57, liver 46), the hindgut (intestine 51, 52) or the anterior foregut (lung 53) lineages. Following this initial treatment, spheroids spontaneously form and upon their transfer to matrigel and organoid culture conditions, are able to form their respective organoids on a time scale similar to the time taken in the formation of anterior structures (See above and Table 1).

The period of time required to generate the correct information for self‐assembly of these organoids may only take place once a suitable signalling niche has been generated by directed differentiation. For example, during the formation of liver organoids from human iPSCs 46, after their differentiation towards definitive endoderm, the cells require co‐culture with Human Umbilical Vein Endothelial Cells (HUVECs) and human mesenchymal stem cells (MSCs) for cell coalescence and liver organoid formation 46; this has marked similarities with the co‐culture of Paneth cells with LGR5^+^ SCs described above 47. In general, multiple rounds of signalling factors may generate the cellular heterogeneities required for symmetry‐breaking events to occur and, as subsequent cell fates are acquired, different genetic programs may be activated over time that facilitate the organization and patterning of the tissues prior to the self‐assembling events.

In summary, a common feature in the examples of anterior and visceral organoids is the generation of complex structures through an initial phase of generation and organization of different cell types which can vary depending on the tissue lineage and whether single SCs are used or isolated sub‐structures of tissues (e.g. intestinal crypts). This initial phase specifies a particular pattern or region before a longer phase of self‐assembly builds upon this pattern. In terms of single cells, they are capable of generating positional information de novo in order to achieve this construction. However, they are not dependent on an external source of a Morphogen (evidenced by the fact that these cultures take place in a signalling environment that is assumed to be uniform) but may be able to generate subsequent lineages that generate and secrete their own patterning factors.

## Gastrulation as a result of symmetry breaking within an ensemble of embryonic stem cells

The process of gastrulation represents a crucial event in animal development as it transforms a mass of similar cells into the physical outline of an organism with recognisable body axes and germ layers (the seeds for the different tissues and organs) 58. This process is common to all animal embryos and is driven by a conserved set of molecular interactions which lay down a transcriptional map upon an otherwise phenotypically similar group of cells 58, 59. This map acts as a cue for a complex choreography in which groups of cells move from the outside to the inside of the embryo to give rise to the mesoderm and the endoderm. The physical implementation of this process depends on the geometry of the embryo. For example, in amphibia it involves the ordered invagination of cell populations through an orifice in a ball of cells, the blastopore 60, while in birds and mammals, gastrulation creates a dynamic longitudinal furrow within an epithelial disc (chicken, rabbit and human) 61, 62, 63 or cylinder (rodents) 64, known as the Primitive Streak. In all cases, cells undergoing gastrulation express the T‐box transcription factor T/Brachyury (T/Bra) 65 and follow an orientation with respect to some global axial system that has been laid down in the embryo. T/Bra integrates spatial and temporal signals at the level of individual cells 66, promotes their movement 66, 67, 68 and, together with those signals, implements specific fates. The outcome of gastrulation is the assignation of different cells to the anteroposterior and dorsoventral axes of the organism and the localization of the endoderm and mesoderm to the inside and the ectoderm to the outside of the embryo, respectively. The detailed spatiotemporal correlation of these processes and their relationship to gene expression highlight how remarkable it is that eye cups 10, 26, intestines 51, 52 and pancreas 40 can emerge from SCs without a coordinate system.

Cells in adherent culture can be made to recapitulate some features associated with gastrulation 66, 69. For example, one of the hallmarks of the process, rapid cell movement associated with transient *T/Bra* gene expression, can be observed in cultures of ESCs differentiating in the presence of Activin/Nodal and Wnt signalling 66. The timing and sequence of these events is very similar to those in the embryo 64, 70. Furthermore, human ESCs arranged on micropatterned discs and induced to differentiate by BMP4 give rise to a radially symmetric pattern of gene expression with an arrangement that attempts to mirror the topological organisation in the embryo 69; from the outside to the inside: extraembryonic (Cdx2), Endoderm (Sox17), mesoderm (T/Bra) and neural (Sox2). This organization would be a flattened projection of the mouse cylinder and also of the arrangement in a human embryo. In this arrangement, the adhesion to the substrate prevents morphogenesis suggesting that it is possible to separate the morphogenetic movements from the fate, a conclusion that had been obtained from experiments in chicken embryos suggesting that the morphogenetic movements are there to make an embryo. Two intriguing observations of the micropatterned hESCs are that the process does not scale and that the pattern appears to use the boundaries as a reference 69.

The radial symmetry of the micropatterned hESCs contrasts with the anisotropic and polarised patterns of activity of cells in early embryos and begs the question of the origin of these asymmetries. One possibility is that these asymmetries require a three dimensional organisation but it may be that they reflect a combination of dimensionality, movement and localised cues. Indeed, in embryos, cells become endowed with such asymmetries very early in development and might be registered in manners that allow them to move (or they are endowed with the ability to move). For example, when explants are made from the region around the organiser of *Xenopus* embryos, the cells undergo polarised convergence extension 71, supporting the notion of an intrinsic “navigation system.” As in the case of the retina from ESCs, not only the fates, but also the mechanical properties of the system are autonomously encoded. This view is clearly demonstrated in experiments in which *Xenopus* animal caps treated with Activin, which if undisturbed would develop into neuroectoderm, will not only turn into mesoderm but will organize themselves into polar structures that resemble the exogastrulae characteristic of Keller sandwiches 71. Furthermore, if one takes these structures that have been exposed to Activin, disaggregates them and lets the cells aggregate, they will form structures that resemble the original ones 72 i.e. the system can reassemble itself. For these reasons it is perhaps not surprising that ESCs can organize themselves into similar structures.

**Figure 3:**
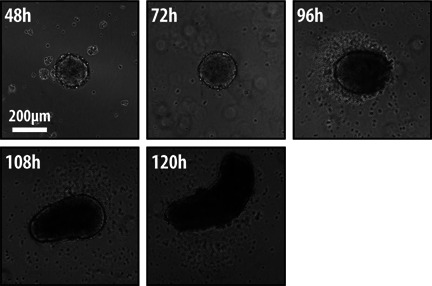
Time‐course describing the formation of a *Gastruloid* over time. Aggregates of small numbers of mESCs plated in low‐adhesion plates will display many of the characteristics of early embryo development such as polarization in gene expression and axial elongation 73, 74. Shown in this figure is the development of a single *Gastruloid* exposed to a pulse of signalling between 48 and 72 hours. Observe the gradual elongation from one region.

Experiments with three dimensional aggregates of mouse ESCs reveal an intrinsic tendency to break symmetry 25, 73, 74. For example, when large EBs are exposed to Wnt signalling, occasionally they exhibit axial organization in the form of an asymmetric activation of Wnt signalling and expression of T/Bra 25; although the causes for this are not known. However, a first glimpse of consistent axial organization of ESCs was reported in P19 Embryo Carcinoma cells 75. When EBs of P19 cells are placed in serum, they organize into structures that very much resemble exogastrulae with a polarised extension that expresses T/Bra in a Wnt‐dependent manner 75. Furthermore, gene expression analysis of these aggregates indicates that they differentiate further and express mesodermal genes much as they would do in the embryo 75. Recently, mouse ESCs have been shown to be able to undergo reproducibly similar patterning events 73, 74 (Fig. 3). Analysis of the cause underlying this behaviour revealed a need for an initial critical cell number and the requirement for Wnt signalling for the elongation 73. In addition, these events are associated with a process that resembles gastrulation, whereby cells move from a defined position and with a direction 73. Furthermore, filming of the emergence of these structures over time shows how the symmetry is broken and how they evolve 73. The development of these structures, called *Gastruloids* (Fig. 3), leads to axial extensions in a manner that parallel similar events in embryos.

In contrast with the adult organoids, *Gastruloids* can be followed over time with good temporal resolution 73. The picture that emerges from these observations is one of cell autonomous molecular processes that unfold over time to produce asymmetry and spatially ordered structures.

## Genetically encoded self‐assembly

There is a widespread view that the ability of stem cells to generate tissues and organs is an example of self‐organization. We believe that such statements confuse self‐organization with self‐assembly (Box 1). For example, in some instances it has been suggested that a criterion for self‐organization is the ability of a system to put itself together after its structure has been disrupted 76. As in these instances there might be a “memory” of the original arrangement in the elements that are produced by the destruction of the original order, this might be better described as an example of self‐assembly. Such processes have been observed in many instances (e.g. vertebrate limbs and insect imaginal discs) and are used as a basis for the assembly of some organs from stem cells in the form of natural scaffolds around which different parts arrange themselves 43. In contrast with these processes, a canonical self‐organizing system achieves dissipative, nonequilibrium order at the global level through local interactions within a collection of its components 77. This can be induced by internal and external factors, but critically order is lost upon removal of the source of energy 77 (see also 27 in the context of biological systems). Classical examples of self‐organizing systems are spontaneous magnetisation 78, lasers 79, the Belousov‐Zhabotinski reaction (classic Turing patterns) 80, 81 and in biology, bird flocking 82. The emergence of organoids does not belong to either of these classes as in the initial phases of the process it is not clear that all the components of the system (i.e. the cells), are equivalent‐populations of SCs, particularly ESCs which are dynamically heterogeneous. While these heterogeneities can be the source of signals that promote patterning in a Turing like mechanism, the ingredients for the final structure are not present in the starting population and emerge in an ordered manner over time. Furthermore, removal of the trigger does not result in the decay of the structure.

**Figure 4:**
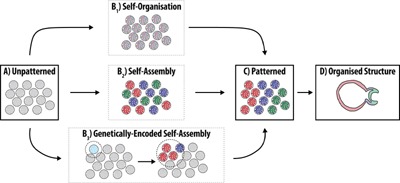
Mechanisms for pattern formation. Groups of cells (large circles), initially displaying no intrinsic pattern (**A**) may form a properly patterned tissue (**C**) through a number of mechanisms (**B_1–3_**), where the pattern serves as a blueprint for the assembly and growth of the tissue to generate a fully organized structure (**D**). In the case of self‐organization (see Fig. 1B), the fluctuations in the expression of genes within individual cells (denoted by red, green and blue colors) eventually allow Turing‐like Reaction‐Diffusion mechanisms to pattern the tissues (**B_1_**). With self‐assembly, cells which have already acquired an identity can undergo a degree of sorting, self‐assembling into the required pattern (**B_2_**). Patterning through genetically encoded self‐assembly requires Turing‐like mechanisms to establish a localised source of signalling (pale blue cell) which, following stabilisation, serves as a reference for the patterning of the tissue (**B_3_**).

The heterogeneities inherent to the organoid systems and the genetic encoding of the process associated with their evolution in culture, make the point that these are not self‐organizing systems in the sense of classical physical systems. In all cases, the biological systems evolve through local interactions that are encoded in their components and unfold over time (see above). The intrinsically encoded governance of the process is an essential element of these systems and leads to what James Briscoe has called “supervised self‐organization” (personal communication). This highlights that while there is an underlying element of progressive self‐organization in the process, this stems from the unfolding of a genetic program within ill‐understood physically constrained conditions (Fig. 4). A related thought has been emphasised by Sasai and colleagues e.g. the curvature of the optic cup and the forces that lead to its symmetry breaking in the emerging structure are autonomous to the structure 26; this is important to understand. It is clear that the genetic programs encode elements that can mediate emergent properties by interacting with the environment, other cells or the mechanics that results from their packing and this encoding and its feedback on the cell autonomous events is, probably, the key to the process. The divergence of programs leads to the generation of interacting sets of cells but the gene expression programs are autonomous. Thus it is possible to observe these programs in the differentiation of ESCs and less clearly in the intestinal organoids. We would suggest that we should talk about *genetically supervised self‐organization* or, perhaps more properly, *genetically encoded self‐assembly* (Fig. 4B_3_) (Box 1).

### Programs of gene activity lead to the autonomous emergence of tissues and organs

An important question from these studies is not only whether the resulting structures are the same as those that emerge in embryos but whether the process that leads to them is the same as that followed by cells in embryos. The emergence of a floor plate (FP) in differentiating neural cysts highlights many of the elements of this discussion 83.

In the embryo, the generation of motorneurons depends on the activity of a source of Shh that is located on the ventral side of the neural tube 84. This pattern is induced early in development by the underlying notochord and strategically positions the neural progenitors next to the somitic mesoderm progenitors of the muscles that they will innervate. Under mechanically and chemically controlled conditions ESCs can be coaxed to differentiate into neuroepithelial structures with a lumen that resembles a neural tube 83. Surprisingly, when treated with Retinoic Acid, a patch of Sonic Hedgehog (Shh)‐producing floor‐plate emerges in around 45% of the cysts. The FP begins to form around three days, after which it matures (as indicated by the expression of ARX at day 7) and patterns the rest of the neural tissue, forming ten distinct layers by day nine. There is no notochord in the culture suggesting that a floor plate is an integral part of the “neural tube genetic program” and that when activated, within the right length scale, cell interactions mediated by signalling molecules, lead to the emergence of a single structure 83.

**Figure 5:**
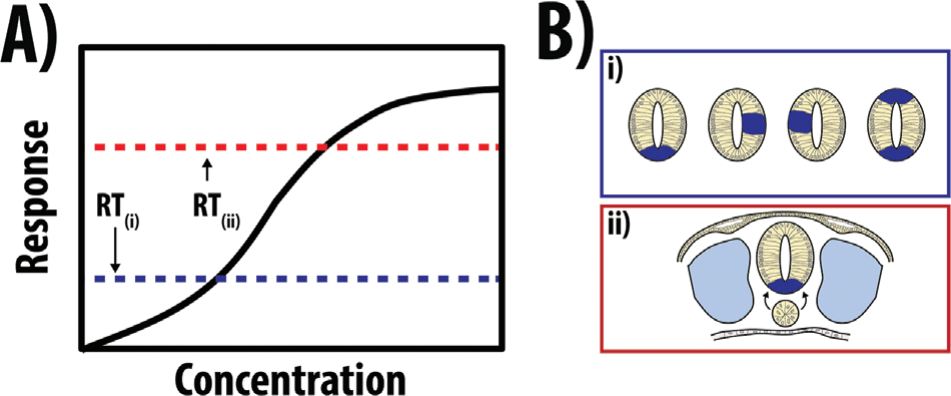
Hypothesis for differences in intrinsic patterning between in vitro and in vivo. The observation that differentiating neural cysts can generate a floor plate in vitro in the absence of a notochord (which acts as the source for Shh‐mediated ventral‐dorsal patterning) may be due to the differences in a threshold level (RT_(i)_, RT_(ii)_) required to initiate an intrinsic patterning event in these cells. **A**: In vitro, if it were possible to accurately determine the axial orientation of these cysts, the floor plate (**Bi**, indicated by dark blue shading) would not be positioned in the same place in different cysts, contrasting with the precise positioning of the floor plate in the embryo (**Bii**). In the case of the embryo, the positioning of the somites, notochord and surrounding tissues serve to restrict the formation of the floor plate to one particular region, effectively raising the threshold for patterning (**Aii, Bii**).

How then is it possible to have such a precise structure autonomously? It may be that in the experimental culture conditions, the threshold for the intrinsic patterning process is very low which allows it to occur with ease (Fig. 5Ai); in the fact that 4/207 cysts presented with two FPs demonstrates that there is the potential to specify more than one site within a cyst 83. If one could provide a fixed axial reference to these cysts, it is likely that the FP would appear in a different position in different cysts (Fig. 5Bi) which contrasts with the situation in the embryo where the FP is always precisely positioned with regard to the somites and, importantly, the notochord (Fig. 5Bii). We surmise that the difference between the embryo and the culture lies in the threshold needed to trigger the emergence of the FP. The culture reveals an intrinsic ability of the system, genetically encoded, to generate a FP with a very low threshold (Fig. 5Ai and Bi) which in the embryo is raised by interactions with the surrounding tissues (Fig. 5Aii and Bii). This means that the FP would only emerge upon a specific signal which is positioned in such a manner that allows motorneurons to be specified where they are needed, and in a reproducible manner.

This example suggests that there are programs of gene activity that lead to the autonomous emergence of tissues and organs i.e. that there might be tissue/organ contained programs (cell autonomy but by encoding emergent properties, they produced tissues). Such self‐contained patterning programs are obvious in holometabolous insects and have been the basis for the understanding of pattern formation in *Drosophila*, where every part of the adult develops from an autonomous self‐patterning structure: an imaginal disc 85. It is these intrinsic programmes that are revealed by the organoids 26.

## The system and the pattern: How genetic programs generate time and space

Summarizing the key elements of a research program in Developmental Biology Viktor Hamburger admonished practitioners that “our real teacher has been and still is the embryo, who is, incidentally, the only teacher who is always right” (cited in 86). Hamburger was an embryologist/developmental biologist and represents a tradition that, in our view, is being challenged by the autonomous patterning of differentiating stem cells. For an embryologist, an embryo is a whole that patterns itself in relation to each of its component parts and the aim of developmental biology is, indeed, to understand how the parts come together to make an embryo – a specific pattern. However, when we do this what we learn is how an embryo, *a* particular embryo from *a* particular species, is put together. For example the way BMP and Chordin are deployed and used to pattern early embryos varies between them, with most dramatic differences between and across phyla 87, 88, 89. In a way, it looks as if every embryo represents a problem that is solved in a specific manner by an intrinsically encoded, and probably conserved, biochemical system. By focusing on specific patterns we might miss the important element of the system: the underlying molecular system. The issue at stake might not be the embryo but the structure of the underlying system that is so conserved and yet so adaptable: genetic programs that are modified and supervised by versatile molecular systems that we know little about. Organoids might represent a way to focus on such systems and their mechanisms: the ones that act autonomously to generate particular organs and systems, and those that integrate them at a higher level.

## Conclusions

There might be surprises ahead as the molecular mechanisms underlying the generation of organoids might be different from those mediating the corresponding organs in vivo. The organoids in their own way represent a new challenge to the molecular systems that underlie pattern formation and we might find that although the final structures are very similar to those produced in embryos, their paths are different. In probing this, we shall learn how to harness the molecular systems to “replicate” tissues and organs in vitro. At this, the interface between *genetically encoded self‐assembled* organoids with designer bioengineering 90 promises much, but the harvesting of this interaction will bring about an interesting reassessment of developmental biology, more focused on molecular mechanisms than on patterns. One of the lessons already learned from these studies is a reconciliation of Turing‐driven mechanisms and Wolpertian positional information 91 as it is clear that the former drives the emergence of localised signalling sources that, when stabilised, act as references for patterning: positional information is a result of genetically encoded self‐assembly. There are more to come.

This research invites a consideration of the moral and ethical issues associated with these structures, in particular the embryonic ones 92, but is imperative that this goes hand in hand with the development of robust and reproducible experimental systems in which we understand the cellular and molecular events that fuel these structures.

The authors declare no conflicts of interest.
